# MicroRNA-21 modulates brown adipose tissue adipogenesis and thermogenesis in a mouse model of polycystic ovary syndrome

**DOI:** 10.1186/s13293-024-00630-2

**Published:** 2024-07-10

**Authors:** Samar Rezq, Alexandra M. Huffman, Jelina Basnet, Amira E. Alsemeh, Jussara M. do Carmo, Licy L. Yanes Cardozo, Damian G. Romero

**Affiliations:** 1 Department of Pharmacology and Toxicology, University of Mississippi Medical Center, 2500 N. State Street, Jackson, MS 39216 USA; 2 Department of Physiology and Biophysics, University of Mississippi Medical Center, 2500 N. State Street, Jackson, MS 39216 USA; 3 Department of Medicine, University of Mississippi Medical Center, 2500 N. State Street, Jackson, MS 39216 USA; 4 Women’s Health Research Center, University of Mississippi Medical Center, 2500 N. State Street, Jackson, MS 39216 USA; 5 Cardiovascular-Renal Research Center, University of Mississippi Medical Center, 2500 N. State Street, Jackson, MS 39216 USA; 6 Cardiorenal and Metabolic Diseases Research Center, University of Mississippi Medical Center, 2500 N. State Street, Jackson, MS 39216 USA; 7 Department of Anatomy, Histology, and Embryology, Faculty of Medicine, Zagazig University, Zagazig, Egypt; 8 Department of Cell and Molecular Biology, University of Mississippi Medical Center, 2500 N. State Street, Jackson, MS 39216 USA

**Keywords:** Polycystic ovary syndrome, Brown adipose tissue, Thermogenesis, Adipogenesis, Energy expenditure

## Abstract

**Background:**

Polycystic ovary syndrome (PCOS), the most common endocrine disorder in premenopausal women, is associated with increased obesity, hyperandrogenism, and altered brown adipose tissue (BAT) thermogenesis. MicroRNAs play critical functions in brown adipocyte differentiation and maintenance. We aim to study the role of microRNA-21 (miR-21) in altered energy homeostasis and BAT thermogenesis in a PCOS mouse model of peripubertal androgen exposure.

**Methods:**

Three-week-old miR-21 knockout (miR21KO) or wild-type (WT) female mice were treated with dihydrotestosterone (DHT) or vehicle for 90 days. Body composition was determined by EchoMRI. Energy expenditure (EE), oxygen consumption (VO2), carbon dioxide production (VCO2), and respiratory exchange ratio (RER) were measured by indirect calorimetry. Androgen receptor (AR), and markers of adipogenesis, de novo lipogenesis, angiogenesis, extracellular matrix remodeling, and thermogenesis were quantified by RT-qPCR and/or Western-blot.

**Results:**

MiR-21 ablation attenuated DHT-mediated increase in body weight while having no effect on fat or BAT mass. MiR-21 ablation attenuated DHT-mediated BAT AR upregulation. MiR-21 ablation did not alter EE; however, miR21KO DHT-treated mice have reduced VO2, VCO2, and RER. MiR-21 ablation reversed DHT-mediated decrease in food intake and increase in sleep time. MiR-21 ablation decreased some adipogenesis (Adipoq, Pparγ, and Cebpβ) and extracellular matrix remodeling (Mmp-9 and Timp-1) markers expression in DHT-treated mice. MiR-21 ablation abolished DHT-mediated increases in thermogenesis markers Cpt1a and Cpt1b, while decreasing CIDE-A expression.

**Conclusions:**

Our findings suggest that BAT miR-21 may play a role in regulating DHT-mediated thermogenic dysfunction in PCOS. Modulation of BAT miR-21 levels could be a novel therapeutic approach for the treatment of PCOS-associated metabolic derangements.

## Highlights

- MicroRNA-21 genetic ablation decreased excess androgen-mediated increase in body weight and lean mass.
- Excess androgens decreased oxygen consumption (VO2), carbon dioxide production (VCO2), and respiratory exchange ratio (RER), without affecting energy expenditure (EE) in microRNA-21 knockout mice.
- MicroRNA-21 genetic ablation decreased the expression of some brown adipose tissue markers of adipogenesis in excess androgen-treated mice.
- MicroRNA-21 genetic ablation decreased the expression of some brown adipose tissue markers of extracellular matrix remodeling in excess androgen-treated mice.
- MicroRNA-21 genetic ablation reversed the androgen-mediated increased expression of some genes related to thermogenesis in brown adipose tissue of androgen-treated mice.

## Plain language summary

Polycystic ovary syndrome (PCOS) is a common hormone disorder in premenopausal women, often linked to obesity and abnormal brown fat tissue activity. Women with PCOS have elevated male hormones, which are responsible for many metabolic problems. Our study focuses on understanding the role of microRNA-21 (miR-21) in the energy balance and brown fat tissue activity in a PCOS mouse model. We studied female mice with and without miR-21, treating them with a male hormone. We measured body composition and energy expenditure. We also analyzed the levels of specific genes and proteins related to fat tissue and energy production. Our findings showed that mice lacking miR-21 had less weight gain in response to male hormones, without fat or brown fat tissue mass changes. They also had reduced energy production, changed eating habits, and altered expression of genes related to fat tissue and energy production. In conclusion, our study suggests that miR-21 in brown fat tissue may regulate the energy imbalance caused by male hormones in PCOS. Adjusting miR-21 levels in brown fat tissue could be a new way to address the metabolic issues associated with PCOS.

## Introduction

Polycystic ovary syndrome (PCOS) is the most common endocrine disorder in reproductive-age women, with a prevalence ranging from 4–21% depending on the diagnostic criteria used [1]. PCOS is characterized by oligo- or anovulation, polycystic ovaries and clinical and/or biochemical signs of hyperandrogenism. Women with PCOS have higher incidence of multiple cardiovascular risk factors such as obesity, elevated blood pressure, renal injury, dyslipidemia, and insulin resistance [2–7]. Obesity is the most common of these risk factors, with the majority of women with PCOS being obese or overweight [8, 9] and having increased central adiposity [10]. Women with PCOS have lower postprandial thermogenesis, which is positively associated with insulin resistance [11]. Moreover, supraclavicular skin temperature is inversely correlated with serum testosterone level in PCOS women [12]; however, the detailed molecular mechanisms involved in androgen-mediated altered thermogenic responses in PCOS are not fully understood.

Brown adipose tissue (BAT) dissipates energy in the form of heat by uncoupling the mitochondrial respiratory chain and ATP synthesis, in contrast to white adipose tissue (WAT), which stores excess energy [13]. WAT and BAT dysfunction contributes to the development of obesity-related metabolic disturbances due to impairment in the regulatory circuits of fuel storage and oxidation [14]. Inducing BAT activity can mitigate obesity-related metabolic dysregulation by increasing energy utilization via thermogenesis [15, 16]. Thus, BAT plays an important role in weight management and metabolic homeostasis.

Thermogenesis in mitochondria-rich brown adipocytes involves a series of steps that begin with the release of free fatty acids (FFAs) by lipolysis and continue with their transport to the mitochondria to undergo β-oxidization by carrier proteins such as fatty acid-binding proteins (FABPs) and carnitine palmitoyltransferase 1 (CPT1) [17, 18]. Uncoupling protein 1 (UCP1) is a proton channel found in the inner mitochondrial membrane that serves to uncouple the mitochondrial electron transport chain from ATP production, directing it to thermogenesis and resulting in the release of significant amounts of chemical energy in the form of heat [19]. Therefore, UCP1 is the main thermogenic protein expressed in the BAT [20]. The BAT’s structure and thermogenic functions are regulated by a number of transcription factors. The members of CCAAT/enhancer-binding protein family (C/EBPs) and peroxisome proliferator-activated receptor gamma (PPARγ) are master transcriptional regulators of both WAT and BAT adipogenesis, which is the differentiation of adipocyte precursors (preadipocytes) to mature adipocytes [21, 22]. The thermogenic function of BAT is primarily regulated by transcription machinery that controls the expression of thermogenic genes in brown adipocytes such as peroxisome proliferative activated receptor gamma coactivator 1 alpha (PGC-1α; gene Ppargc1a) [23], PR domain containing 16 (PRDM16) [24], and cell-death inducing DNA fragmentation factor-like effector A (CIDE-A) [25]. Type 2 deiodinase (DIO2) is an important thermogenic marker that catalyzes the formation of thyroid hormone triiodothyronine (T3) from thyroxine [26], which is one of the major mechanisms controlling BAT activity by inducing mitochondrial biogenesis [27].

Dysregulation of many microRNAs (miRNAs) in metabolic tissues, including the adipose tissue, contributes to the development of obesity and its associated complications [28, 29]. MiRNAs are small, non-coding RNAs composed of 21–25 nucleotides that suppress gene expression at the posttranscriptional level via mRNA degradation or translational repression [30]. Several miRNAs modulate BAT thermogenic activity by regulating brown fat lineage determination and differentiation in a positive or negative manner [31, 32]. One miRNA of particular interest in both PCOS and obesity is miRNA-21 (miR-21). Higher levels of miR-21 are found in the WAT of obese rodents and humans [33, 34]. Furthermore, miR-21 mimic induces thermogenic gene expression in both WAT and BAT and alleviates high-fat diet-induced obesity in mice [35]. Moreover, circulating miR-21 is increased in women with PCOS [36–39]. However, the role of miR-21 in androgen-induced adipose tissue dysfunction and altered thermogenic responses in PCOS is still unknown.

Effective BAT thermogenic machinery is associated with favorable metabolic phenotypes and efficient energy expenditure [20]. Therefore, strategies aimed at increasing BAT activity and thermogenic function may be an effective therapeutic approach for attenuating the metabolic derangements observed in PCOS and other metabolic disorders with increased adiposity, such as fatty liver disease and type 2 diabetes. We aim to test the hypothesis that miR-21 controls brown adipocyte differentiation and adaptive thermogenesis in BAT by targeting key regulators of both adipogenesis and thermogenesis in a mouse model of PCOS. Our research is highly clinically relevant because it adds to our understanding of the multifunctional roles that miR-21 plays in metabolic pathways regulation by identifying its effect on the regulation of BAT thermogenic function in PCOS.

## Materials and methods

### Animals

The generation of miR-21 knock out (miR21KO) mice has been previously reported [40]. Mice were backcrossed for more than ten generations on a C57BL/6N genetic background (Charles River Laboratories, Wilmington, MA). A heterozygous colony was maintained to generate both miR21KO and WT mice littermates for the studies. The animals were kept in standard housing conditions with temperature and humidity control and a 12:12-h light–dark cycle. The mice were fed standard chow (Teklad diet 2018) and water on an ad libitum basis. The experimental protocols were approved by the Institutional Animal Care and Use Committee of the University of Mississippi Medical Center and were performed in accordance with the National Institutes of Health’s Guide for the Care and Use of Laboratory Animal 8th edition (2011).

### Experimental design

PCOS was induced in three-week-old female miR21KO or their wild-type (WT) littermate controls following the method of Caldwell, et al. [41] and as reported in our previous studies [42, 43]. MiR21KO and WT mice were randomly assigned to be implanted subcutaneously with Silastic brand tubes (length, 1.5-cm; id, 1.47 mm; od, 1.95 mm, Dow Corning Corp, catalog no. 508-006) that were either empty or filled with dihydrotestosterone (DHT, Steraloids Inc., Newport, RI, 8 mg) (N = 10/group) and followed up for 90 days. The body weight was measured on a weekly basis. Body composition (lean and fat mass) was assessed with EchoMRI (4in1-EF-016 model Body Composition Analyzer; EchoMRI, Houston, TX) at the end of the experimental period. At the end of the experiment, blood was collected via cardiac puncture, followed by saline perfusion under isoflurane gas anesthesia. The BAT depot was harvested, and a section was flash frozen in liquid nitrogen and stored at -80 °C until further processing. Another BAT section was fixed in 10% neutral buffered formalin for histological examination.

### Energy expenditure, O_2_ consumption, CO_2_ production, motor activity, and sleep time

To assess energy expenditure (EE), an additional set of 12-week old mice (N = 6–10/group) were placed individually in metabolic cages connected with a computer-controlled indirect calorimetry system (Promethion Metabolic Measurement System, Sable International, North Las Vegas, NV) equipped with gas sensors to continuously measure oxygen consumption (VO2) and carbon dioxide production (VCO2) for 6 consecutive days. The respiratory exchange ratio (RER), which is a measure of the predominant fuel source, was calculated as the VCO2/VO2 ratio. The locomotor activity was determined based on the number of infrared light beams breaks mounted in the cages in X, Y, and Z axes, and the total distance the animals moved in the cage was measured in meters. The sleep time, defined as the animal's immobility for more than 40 s, was recorded. Data acquisition and raw data processing were performed by MetaScreen v2.3.15 and ExpeData v1.8.4. Data analysis was performed using the web-based analysis tool for indirect calorimetry experiments CalR v1.2 [44].

### mRNA expression of adipogenesis, thermogenesis, and ECM remodeling genes

BAT total RNA (N = 6 mice/group) was extracted, followed by DNAse treatment, quantification, and reverse transcription, as previously described [42, 45]. TaqMan gene expression assays (Thermo Scientific, Waltham, MA) were used to perform quantitative RT-qPCR for adipogenesis, thermogenesis, and ECM remodeling markers (Table 1). Additionally, isoforms of the electron transport chain component cytochrome c oxidase, a key thermogenesis marker reported to be modulated by several miRNAs [46–48], and Elovl3 gene, a member of the fatty acid chain elongase family, likely involved in thermogenesis and brown fat recruitment [49], were also assessed (Table 1). RT-qPCR reactions were performed under fast run conditions (50 °C for 2 min, 95 °C for 20 s, followed by 40 cycles of 95 °C for 1 s and 60 °C for 20 s) using Luna Universal Probe qPCR Master Mix (New England Biolabs, Ipswich, MA) in an Applied Biosystems QuantStudio 3 thermal cycler (Thermo Scientific). qPCR product quantification was performed by the ∆∆Ct quantification method, and expressed as arbitrary units (AU) standardized against the geometric mean (GM) of the reference genes (Actb, B2m, Gapdh, and 18S rRNA).

**Table 1:** TaqMan gene expression assays for RT-qPCR of adipogenesis and thermogenesis target genes

Gene symbol	Assay ID
Actb	Mm02619580_g
Adipoq	Mm00456425_m1
AR	Mm00442688_m1
B2m	Mm00437762_m1
Cebpb	Mm00843434_s1
Cidea	Mm00432554_m1
Cpt1a	Mm01231183_m1
Cpt1b	Mm00487191_g1
Cox4i1	Mm01250094_m1
Cox4i2	Mm00446387_m1
Cox7a1	Mm00438297_g1
Cox8b	Mm00432648_m1
Dio2	Mm00515664_m1
Elovl3	Mm00468164_m1
Gapdh	Mm99999915_g1
Mmp9	Mm00442991_m1
Pparg	Mm00440940_m1
Ppargc1a	Mm01208835_m1
Prdm16	Mm00712556_m1
18S	Hs99999901_s1
Timp1	Mm01341361_m1
Ucp1	Mm01244861_m1
Vegfa	Mm00437306_m1

### miRNA-21 quantification

Following the RNA extraction and DNAse treatment described above, miR-21 expression was quantified in BAT tissue (N = 6 mice/group). The reverse transcription was carried out with 10 ng total RNA using the miRCURY LNA RT kit (Qiagen, Germantown, MD). The miR-21 RT-qPCR reaction was performed in a 10 μL final reaction volume including 3 μL of diluted cDNA (1:60), miRCURY LNA SYBR Green master mix, and miRCURY LNA PCR primers for mmu-miR21-5p (Qiagen) in a QuantStudio 3 thermal cycler. qPCR product quantification was performed by the ∆∆Ct quantification method, and expressed as arbitrary units (AU) standardized against the reference gene SNORD110 (Qiagen).

### Western-blot analysis

Brown adipose tissue (N = 4 mice/group) was homogenized in RIPA buffer containing Halt protease and phosphatase inhibitor cocktail (Thermo Scientific). The total protein concentration was quantified with the bicinchoninic acid protein assay kit (Thermo Scientific). Fifty micrograms of total protein were resolved by SDS-PAGE and transferred to Immobilion-P PVDF membranes (Millipore, Burlington, MA). The membranes were blocked with 5% non-fat dry milk in Tris-buffered saline containing 0.1% Tween 20 for 1 h at room temperature, and then incubated overnight at 4 °C with the following primary antibodies: Androgen Receptor (AR, 1:1000; Abcam ab133273, Cambridge, MA, USA); UCP1 (1:1000; Cell Signaling Technology 14670); CPT1A (1:1000; Cell Signaling Technology 12252, Danvers, MA, USA); CPT1B (1:1000; Proteintech 12252, Rosemont, IL, USA); Adiponectin (1:1000; Cell Signaling Technology 2789); cytochrome c oxidase (COX4) (1:1000, Cell Signaling Technology 4844); DIO2 (1:1000; Abcam ab77779); CIDEA (1:2,000; Abcam ab8402); phospho-C/EBPα (Ser21) (1:1000; Cell Signaling Technology 2841); C/EBPα (1:1000; Cell Signaling Technology 8178); phospho-C/EBPβ (Thr235) (1:1000; Cell Signaling Technology 3084S); C/EBPβ (LAP) (1:1000; Cell Signaling Technology 3087); Acetyl-CoA carboxylase (ACC) (1:5000; Cell Signaling Technology 3676); Fatty acid synthase (FAS) (1:5000; Cell Signaling Technology 3180); FABP4 (1:3000; Cell Signaling Technology 2120); PPARγ (1:2000; Cell Signaling Technology 2435); Perilipin-1 (1:5000; Cell Signaling Technology 9349); or GAPDH (1:3,000,000; Cell Signaling Technology 5174). The membranes were incubated with horseradish peroxidase-conjugated goat anti-rabbit or anti-mouse IgG secondary antibodies (1:10,000; Jackson Immuno Research 111–035–003 or 115–035–003) for 1 h at room temperature. Chemiluminescence was detected with SuperSignal West Pico PLUS kit (Thermo Scientific). The ChemiDoc MP imaging system (Bio-Rad, Hercules, CA) and ImageJ software (National Institutes of Health) were used to capture and quantify the images, respectively.

### Histopathological examination

BAT specimens (N = 4 mice/group) were fixed in 10% neutral buffered formalin and paraffin embedded. Tissue sections (5 µm thick) were stained with hematoxylin and eosin (H&E) and imaged with an Olympus DP80 camera mounted on a BX63 motorized microscope. Tissue sections were analyzed by a pathologist (A.E.A.) blinded to the animal groups.

### Statistical analysis

The results are presented as mean ± SEM. Statistical analysis was performed using two-way ANOVA followed by Fisher’s LSD test. Statistical calculations were conducted with GraphPad Prism Software (GraphPad Software, Inc., version 8.4.3). Differences between groups were considered significant at P ≤ 0.05.

## Results

### DHT and miR-21 effects on BAT mass and body composition

**Fig. 1:**
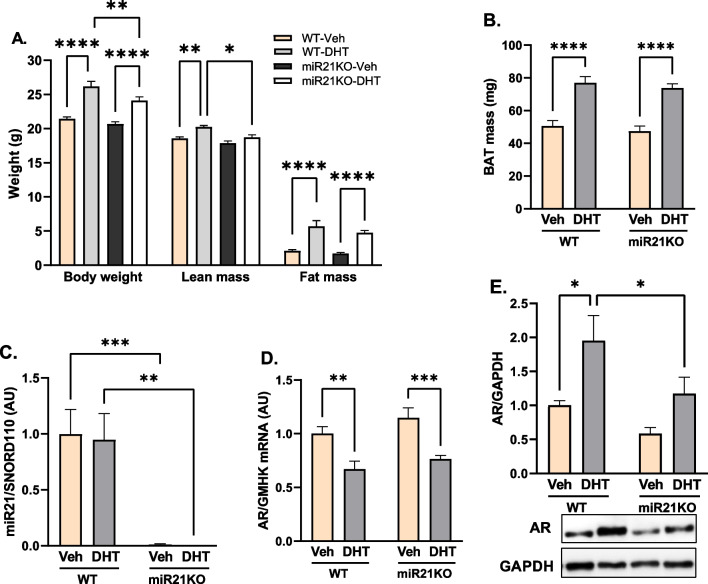
Effect of miR-21 on DHT-mediated effects in body composition and BAT miR-21 and AR expression. WT or miR21KO mice were treated with dihydrotestosterone (DHT) or vehicle (Veh) for 90 days. **A** Body weight was determined by gravimetry, fat and lean masses were determined by EchoMRI. N = 6/group. **B** BAT mass was determined by gravimetry. N = 6/group. **C** BAT miR-21 was quantified by RT-qPCR, and expressed as arbitrary units (AU) standardized against SNORD110. N = 6/group. **D** BAT AR miRNA was quantified by RT-qPCR, and expressed as AU standardized to the geometric mean of four reference genes (GMHK). N = 4/group. **E** BAT AR protein expression was quantified by Western-blot normalized to GAPDH. N = 6 mice/group. Data are expressed as mean ± SEM. Data were analyzed by two-way ANOVA followed by Fisher’s LSD test. *p < 0.05; **p < 0.01; ***p < 0.001; ****p < 0.0001

After 90 days of DHT treatment, WT-DHT mice had significantly higher body weight (26.16 ± 0.76 vs. 21.44 ± 0.26 g), lean mass (20.24 ± 0.20 vs. 18.57 ± 0.23 g), and fat mass (5.70 ± 0.84 vs. 2.12 ± 0.17 g) compared with WT-Veh mice (Fig. 1A). Additionally, WT-DHT mice show a 1.52-fold increase in the BAT mass (Fig. 1B). Notably, miR21-KO-DHT mice showed attenuated DHT-mediated increase in the body weight, and lean mass while they still have similar increases in both fat mass and BAT mass compared with WT-DHT mice (Fig. 1A, B).

### MiR-21 modulated DHT-mediated AR upregulation in the BAT

To assess the effect of DHT on BAT miR-21 and AR levels, BAT miR-21 and AR expression levels were measured. DHT had no effect on BAT miR-21 expression in WT mice, as shown in Fig. 1C. DHT treatment, on the other hand, resulted in a significant decrease in BAT AR mRNA expression in both WT and miR21KO mice while significantly increasing AR protein levels only in WT mice (Fig. 1D, E). Interestingly, miR-21 genetic ablation had no effect on AR expression at the mRNA level, but it attenuated DHT-mediated AR protein expression increase compared to DHT-treated WT mice.

### Altered energy homeostasis by DHT and its modulation by miR-21

**Fig. 2:**
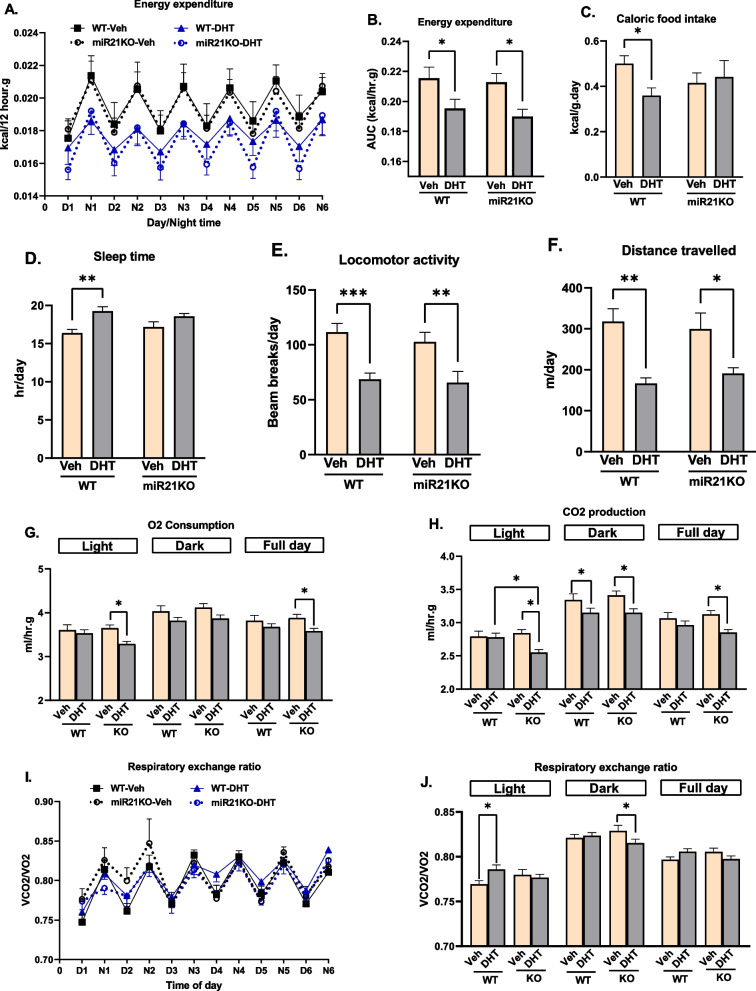
Effect of miR-21 on DHT-mediated changes in energy homeostasis, locomotion and sleep. WT or miR21KO mice were treated with dihydrotestosterone (DHT) or vehicle (Veh) for 90 days. **A**, **B** Energy expenditure (EE) was determined by indirect calorimetry (N = 6–7/group). **C** Food intake is expressed as Kcal and normalized by body weight (N = 6–8/group). **D** Sleep time, defined as the animal's immobility for more than 40 s, was measured in hr (N = 7–10/group). **E**, **F** Locomotor activity (**E**) was determined based on the number of infrared light beams breaks mounted in the cages in X, Y, and Z axes, and the total distance the animals moved in the cage (**F**) was measured in meters (N = 5–8/group). **G**, **H** Oxygen consumption (VO2) (**G**) and carbon dioxide production (VCO2) (H) was measured as the volume of gas produced/consumed/hr and normalized by body weight (N = 6–7/group). **I**, **J** The respiratory exchange ratio (RER) was calculated as the VCO2/VO2 ratio (N = 6–7/group). Data are expressed as mean ± SEM. Data were analyzed by two-way ANOVA followed by Fisher’s LSD test. *p < 0.05; **p < 0.01; ***p < 0.001

We then studied the effect of miR-21 and excess androgens on energy metabolism and activity. As shown in Fig. 2A, B, the increase in fat mass following chronic DHT treatment was accompanied by a significant decrease in energy expenditure (EE) in both genotypes. DHT significantly reduced caloric food intake in WT-DHT mice, whereas no change in caloric food intake was observed in miR21KO-DHT mice (Fig. 2C). DHT increased sleep time in WT mice while it did not have an effect in miR21KO mice (Fig. 2D). DHT decreased motor activity to a similar extent in both mouse strains, as evidenced by a decrease in both locomotor activity (Fig. 2E) and total distance travelled (Fig. 2F). Regarding oxygen consumption, only miR21KO mice had significantly lower full-day and light-phase O2 consumption in response to DHT (Fig. 2G). Moreover, CO2 production was significantly lower in both strains in the dark phase, but only miR21KO-DHT showed a significant reduction in CO2 production in the light phase when compared to miR21KO-Veh and WT-DHT mice (Fig. 2H). The respiratory exchange ratio (RER, VCO2/VO2) was increased in WT-DHT during the light phase but decreased in miR21KO-DHT during the dark phase, suggesting enhanced utilization of carbohydrates as an energy source in WT-DHT mice during the light phase and increased utilization of fats in miR21KO-DHT mice during the dark phase (Fig. 2I, J).

### DHT and miR-21 effects on BAT adipogenesis and de novo lipogenesis

**Fig. 3:**
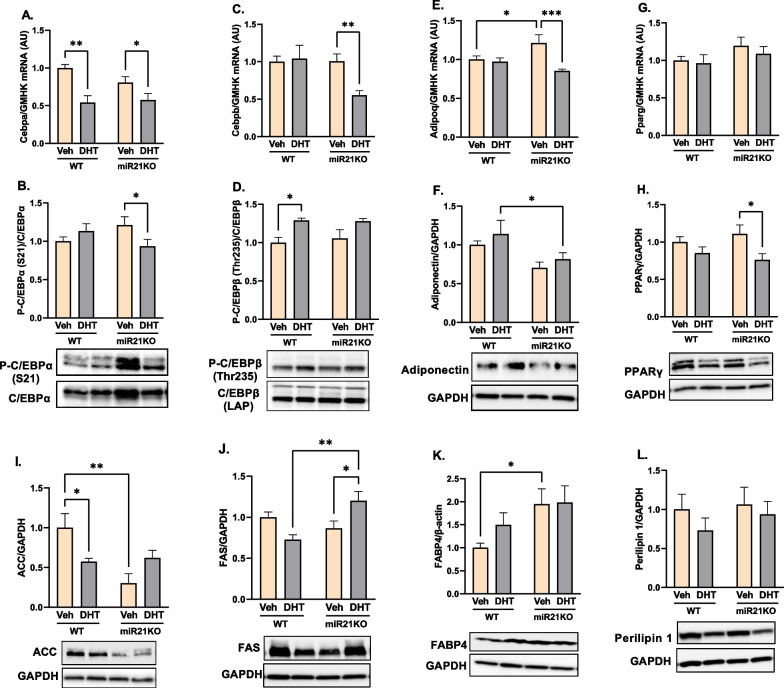
DHT-mediated changes in BAT adipogenesis and de novo lipogenesis are modulated by miR-21. WT or miR21KO mice were treated with dihydrotestosterone (DHT) or vehicle (Veh) for 90 days. C/EBPα (Cebpa, **A**), C/EBPβ (Cebpb, **C**), Adiponectin (Adipoq, **E**), and PPARγ (pparg, **G**) mRNA was quantified by RT-qPCR and standardized to the geometric mean of four reference genes (HGMK) (N = 6/group). C/EBPα (**B**), C/EBPβ (**D**), Adiponectin (**F**), PPARγ (**H**), ACC (**I**), FAS (**J**), Fabp4 (**K**), and Perilipin 1 (**L**) protein was quantified by Western-blot and normalized to GAPDH (N = 4 mice/group). Data are expressed as mean ± SEM. Data were analyzed by two-way ANOVA followed by Fisher’s LSD test. *p < 0.05; **p < 0.01; ***p < 0.001. *ACC* acetyl-CoA carboxylase, *FAS* fatty acid synthase, *Fabp4* fatty acid binding protein 4

To study the interaction between androgens and miR-21 on BAT adipogenesis and de novo lipogenesis, we quantified markers of those processes. As shown in Fig. 3, DHT treatment decreased C/EBPα mRNA (Cebpa) and increased phosphorylated C/EBPβ protein expression in WT mice (Fig. 3A–D), while it did not result in any significant changes in the expression of the RNA and protein of adiponectin (adipoq) and PPARγ (pparg) adipogenesis markers in WT mice (Fig. 3E–H). On the other hand, DHT administration decreased C/EBPα, C/EBPβ, and adiponectin mRNA levels, as well as C/EBPα and PPARγ protein expression in miR21KO mice (Fig. 3A–H). Interestingly, miR21KO-DHT mice had lower levels of adiponectin mRNA in the BAT compared to miR21KO-Veh controls (Fig. 3E) and also have lower levels of adiponectin protein than their WT-DHT littermates (Fig. 3F). MiR-21 genetic ablation abolished the DHT-mediated increases in C/EBPβ phosphorylation observed in WT mice (Fig. 3D). Notably, DHT downregulated acetyl-CoA carboxylase (ACC) protein in WT mice, an effect that was lost in miR21KO mice (Fig. 3I). DHT upregulated fatty acid synthase (FAS) protein only in miR21KO mice with no effect in WT mice (Fig. 3J). While no changes in FABP4 protein levels were observed in both the WT and miR21KO mice in response to DHT, its basal levels were increased in miR21KO-Veh mice (Fig. 3K). The protein expression of perilipin-1, the main lipid droplet-coating protein in adipocytes, did not differ significantly across groups (Fig. 3L).

### MiR-21 genetic ablation modulated adaptive thermogenesis markers

**Fig. 4:**
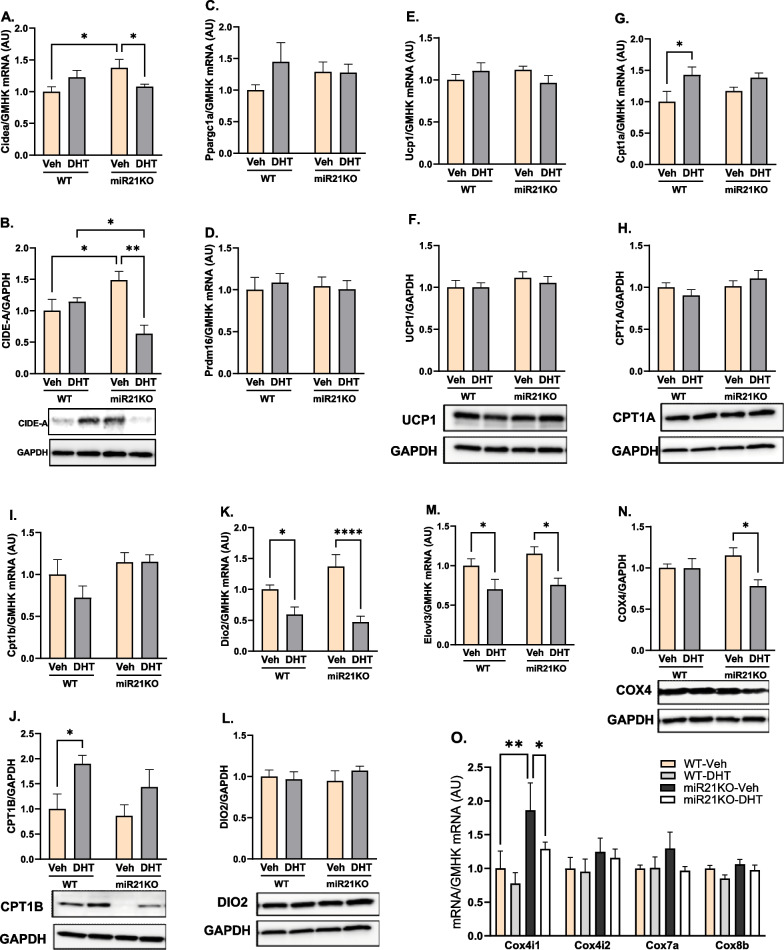
MiR-21 modulates DHT-mediated changes in BAT adaptive thermogenesis targets. WT or miR21KO mice were treated with dihydrotestosterone (DHT) or vehicle (Veh) for 90 days. Cidea (**A**), Ppargc1a (**B**), and Prdm16 (**C**), Ucp-1 (**E**), Cpt1a (**G**), Cpt1b (**I**), Dio2 (**K**), Elovl3 (**M**), and Cox subunits (4i1, 4i2, 7a, and 8b) (**O**) mRNA was quantified by RT-qPCR and standardized to the geometric mean of four reference genes (HGMK) (N = 6/group). CIDE-A (**B**), UCP-1 (**F**), CPT1A (**H**), CPT1B (**J**), DIO2 (**L**), and COX4 (N) proteins were quantified by Western-blot and normalized to GAPDH (N = 4 mice /group). Data are expressed as mean ± SEM. Data were analyzed by two-way ANOVA followed by Fisher’s LSD test. *p < 0.05; **p < 0.01; ****p < 0.0001. *CPT1* carnitine palmitoyltransferase 1, *COX* cytochrome oxidase C, *DIO2* deiodinase 2

To investigate the role of androgens and miR-21 in altered thermogenic responses in PCOS, we assessed several transcriptional cofactors involved in BAT UCP1 expression and mitochondrial biogenesis, including CIDE-A (Cidea), PGC-1α (ppargc1a), and PRDM16. DHT did not modify CIDE-A mRNA or protein in WT mice; however, DHT significantly decreased both CIDE-A mRNA and protein in miR21KO mice (Fig. 4A, B). Moreover, miR-21 genetic ablation per se increased both CIDE-A mRNA and protein expression in miR21KO-Veh compared with WT-Veh mice (Fig. 4A, B). Notably, neither DHT treatment nor miR-21 genetic ablation modified PGC-1α or PRDM16 mRNA expression (Fig. 4C, D).

UCP1 was highly expressed in BAT, but it was not regulated by DHT treatment or miR-21 genetic ablation at the mRNA or protein levels (Fig. 4E, F). DHT treatment upregulated CPT1A mRNA, but not CPT1A protein, in WT mice, an effect that was lost upon miR-21 genetic ablation (Fig. 4G, H). DHT treatment upregulated CPT1B protein, but not CPT1B mRNA, in WT mice, an effect that was lost upon miR-21 genetic ablation (Fig. 4I, J). DHT treatment downregulated Dio2 and Elovl3 mRNA expression to a similar extent in both mouse strains (Fig. 4K–M).

To study the role of excess androgens and miR-21 on BAT mitochondria energetics, we quantified multiple electron transport genes in BAT mitochondria. Neither DHT nor miR-21 genetic ablation had an effect on the gene expression of the brown-adipocyte enriched mitochondrial specific markers COX subunit VIIa1 (Cox7a1) and VIIIb (Cox8b) (Fig. 4O). Moreover, COX subunit IV (Cox4i1 and Cox4i2) mRNA isoforms were not regulated by DHT in WT mice (Fig. 4O). Notably, miR-21 genetic ablation increased Cox4i1 mRNA expression in miR21KO-Veh mice (Fig. 4O). Furthermore, DHT downregulated Cox4i1 mRNA and protein expression exclusively in miR21KO mice (Fig. 4N, O).

### DHT and miR-21 effects on BAT angiogenesis and extracellular matrix remodeling markers

**Fig. 5:**
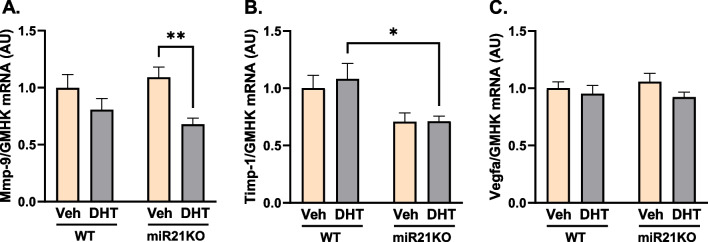
Effect of DHT and miR-21 on BAT extracellular matrix remodeling and angiogenesis. WT or miR21KO mice were treated with dihydrotestosterone (DHT) or vehicle (Veh) for 90 days. BAT gene expression of Mmp-9 (**A**), Timp-1 (**B**), and Vegfa (**C**) was quantified by RT-qPCR and expressed as arbitrary unite (AU) standardized to the geometric mean of four reference genes (GMHK). Data are expressed as mean ± SEM (N = 6/group). Data were analyzed by two-way ANOVA followed by Fisher’s LSD test. *p < 0.05; **p < 0.01. *Mmp-9* matrix metalloproteinase-9, *Timp-1* tissue inhibitor of metalloproteinase-1, *Vegfa* vascular endothelial growth factor-A

To study the interaction between androgens and miR-21 on BAT angiogenesis, and extracellular matrix (ECM) remodeling, we quantified markers of those processes. DHT treatment did not modify the mRNA expression of vascular endothelial growth factor-A (VEGF-A), a key angiogenesis factor that positively regulates BAT thermogenic function [50], and ECM remodeling markers MMP9 and Timp1 in WT mice (Fig. 5A–C). On the other hand, DHT treatment decreased MMP9 mRNA expression in miR21KO mice (Fig. 5A). Notably, while miR-21 genetic ablation had no effect on VEGF-A (Vegfa) expression (Fig. 5C), Timp1 mRNA levels were significantly lower in DHT-treated miR21-KO mice compared with WT littermates (Fig. 5B).

### DHT and miR-21 effects on BAT histopathology

**Fig. 6:**
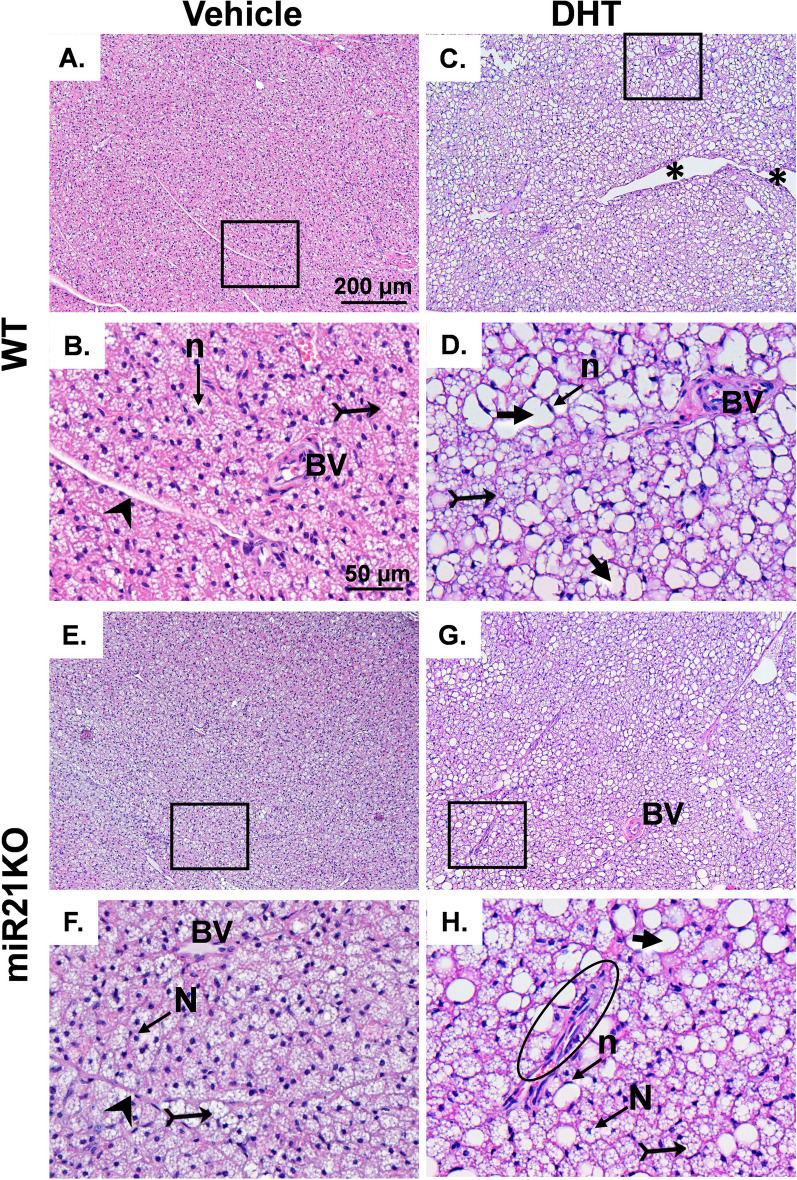
Effect of DHT and miR-21 on BAT histology. WT (**A**, **C**) or miR21KO (**E**, **G**) mice were treated with dihydrotestosterone (DHT) or vehicle (Veh) for 90 days. BAT sections were H&E stained and imaged. **B**, **D**, **F**, and **H** represent higher magnification of the regions (rectangles) in **A**, **C**, **E**, and **G**, respectively. The histopathological features are indicated as follows: Bifid arrow: small lipid droplets, arrowhead: connective tissue septa, circle: disorganized brown adipose tissues, short arrow: single large lipid droplet with irregular outline, and *: wide separation in certain tissue areas. Scale bar for **A**, **C**, **E**, and **G** = 200 μm, Scale bar for **B**, **D**, **F**, and **G** = 50 μm. *BV* normal blood vessel, *BV dilated and congested blood vessel, *n* peripheral signet nucleus, *N* central nucleus

To study the effect of excess androgens and miR-21 at the cellular and tissular level, we performed a histological analysis of BAT tissues. H&E staining of BAT from both WT-Veh and miR21KO-Veh revealed normal brown adipose tissue organization with multiple smaller lipid vacuoles with central nucleus separated by connective tissue septa with normal blood vessels (Fig. 6A, B, E, F). However, BAT sections from WT-DHT mice revealed a disorganized BAT morphology with wide separation in some areas, coleuses of adipocyte’s lipid droplets that appear as a single large lipid droplet with an irregular outline, and a peripheral signet ring nucleus (Fig. 6C, D). Furthermore, there were ill-defined connective tissue septa with multiple fibroblast infiltrations and dilated congested blood vessels. Similar pathological features were observed in BAT sections obtained from miR21KO-DHT mice (Fig. 6G, H). However, fewer coleuses of adipocyte’s droplets were observed in miR21KO-DHT mice when compared to WT-DHT mice (Fig. 6C, D, G, H).

## Discussion

Obesity constitutes a major cardiovascular risk factor in women with PCOS [51]. Expansion of both WAT and BAT due to obesity is reported to be associated with impaired thermogenic responses in humans and animal models [14, 52, 53]. Despite similar resting energy expenditure in PCOS subjects and controls, reduced postprandial thermogenesis has been reported in both lean and obese subjects [11], indicating a possible role of androgens in altered thermogenic activity in PCOS that is independent of obesity. However, the detailed molecular mechanisms involved in this effect are not fully understood. Several miRNAs have been shown to be involved in the regulation of BAT structure and thermogenic function [31, 32]. Increased BAT thermogenic function is associated with favorable metabolic outcomes; consequently, BAT activation or transplant has been proposed as an anti-obesity therapy, and exciting studies in rodents and humans have shown beneficial effects in obesity and glucose homeostasis [54–57]. Moreover, BAT transplant or cold-mediated activation improved multiple reproductive and metabolic traits in rodent models of PCOS [58–62]. Furthermore, combined WAT, BAT, and brain AR genetic ablation protects against androgen excess-mediated obesity and hepatic steatosis in a mouse model of PCOS [63]. Therefore, targeting specific miRNAs to increase BAT function could be an effective therapeutic approach. MiR-21, which is increased in the circulation of PCOS women [36, 37], is upregulated by obesity in brown and white primary adipocytes, as well as subcutaneous and epididymal WAT [33, 34]. Furthermore miR-21 mimic induces the expression of genes involved in browning and thermogenesis in 3T3-L1 adipocytes and the BAT of obese mice [35]. Based on these premises, we performed an exhaustive study of the role of miR-21 whole body energy homeostasis and BAT molecular and histological changes triggered by excess androgens in a well-stablished mouse model of PCOS [64].

The most significant findings in our study of the role of miR-21 in a mouse model of PCOS are: (1) miR-21 genetic ablation mitigated DHT-mediated increase in body weight and lean mass, but it did not modify DHT-mediated increase in fat or BAT mass, (2) miR-21 genetic ablation attenuated DHT-mediated increase in BAT AR protein expression, (3) DHT treatment of miR21KO mice decreased O2 consumption, CO2 production, and RER, without affecting EE, (4) miR-21 genetic ablation abolished DHT-mediated decrease in caloric food intake, (5) miR-21 genetic ablation abolished DHT-mediated increase in sleep time, (6) miR-21 genetic ablation decreased the expression of some adipogenesis markers in BAT of DHT-treated mice, (7) miR-21 genetic ablation decreased the expression of some extracellular matrix remodeling markers in BAT of DHT-treated mice, (8) miR-21 genetic ablation reversed DHT-mediated increases in the expression of some genes related to thermogenesis in BAT.

There are two main types of adipose tissues in mammals: WAT and BAT. BAT is a developmentally formed adipose depot and is prominent in the interscapular region of rodent and human infants. Classical interscapular brown adipocytes are derived from myogenic Myf5-positive precursors and has high mitochondrial density and expression of thermogenic genes [20]. Brown adipocytes are also characterized by the presence of small multilocular lipid droplets serving as fuel reservoirs for mitochondrial respiration. The WAT, on the other hand, has low mitochondrial density, low expression of thermogenic proteins, and is characterized by adipocytes with a single large lipid droplet. Thereby, the main function of WAT is to store excess energy in the form of triglycerides, while the main function of BAT, on the other hand, is to mediate non-shivering thermogenesis and to dissipate excess fuel energy as heat. Therefore, BAT leads the physiological adaptation to cold and whole-body energy homeostasis [65].

The imbalance in food storage and energy expenditure associated with adipose tissue dysfunction is a major mechanism of the metabolic disturbances induced by obesity [14]. Weight gain and increased central/total body fat mass are key clinical features that characterize the majority of women with PCOS and animal models of hyperandrogenemia [10, 66–69]. According to the Androgen Excess-PCOS Society (AE-PCOS), PCOS should be first considered a disorder of androgen excess or hyperandrogenism [70]. Hyperandrogenism is present in more than 80% of women with PCOS, and those women have a worse cardiovascular and metabolic profile than normoandrogenic PCOS subjects [1, 71–73]. In the current study, female mice treated with the non-aromatizable androgen DHT from both miR21KO and WT strains had significantly higher body weight, fat mass, and BAT mass compared to their vehicle-treated littermates. The increase in body weight was attenuated in miR21KO mice due to the decrease in lean mass. BAT miR-21 expression was not modulated in response to DHT in mice in our study. Notably, androgens suppress miR-21 expression in breast cancer cells [74], whereas they increase its expression in LNCaP prostate cancer cells [75]. The findings of those and our studies suggest that androgens have a cell- and/or sex-specific effect on miR-21 expression.

In PCOS women, circulatory miRNAs, including miR-21, are differentially expressed compared to control subjects [36–39]. Moreover, androgens upregulate endometrial AR expression [76]. Our data show that the effects of DHT on body composition were associated with an increase in BAT AR protein, which was reduced in miR21KO mice, indicating that miR-21 has a positive regulatory effect on BAT AR expression. Interestingly, miR-21 can either negatively or positively regulate AR expression depending on the disease or tissue. While miR-21-3p mimics reduce AR expression in cardiac fibroblasts in a diabetic cardiac fibrosis model in male rats [77], miR-21 mimics upregulate AR protein expression in prostate cancer cells [78].

The obesogenic effect of DHT in female mice that we observed was associated with a similar reduction in EE and activity, which was represented by the significant reduction in the locomotor activity and total distance traveled in both genotypes. Androgen administration in male mice was found to decrease locomotor activity despite favorable effects on thermogenic genes expression in skeletal muscle [79]. Notably, androgen-mediated increase in sleep time in DHT-treated mice was attenuated by miR-21 genetic ablation. Women with PCOS experience longer daytime sleepiness, among other sleep disorders, compared to control individuals, likely due to increased melatonin secretion or poor mental health profile [80]. Several miRNAs have recently been proposed to control sleep–wake regulation in humans and animal experimental models [81, 82]. The lack of an effect of miR-21 on EE and locomotor activity suggests that miR-21-mediated sleep time modulation is likely due to central sleep behavioral effects rather than its effect on energy balance.

Interestingly, caloric food intake was reduced by DHT only in WT mice, while it was not affected by DHT in miR21KO mice. Energy balance is maintained when energy from food intake is equal to energy expenditure; moreover, caloric restriction is a potential strategy to counteract excessive weight gain in obesity [83]. Increased adiposity leads to the release of endocrine signals that exert negative feedback in the brain to decrease food intake in conditions of energy surplus [84, 85], hence the decreased caloric food intake in DHT-treated WT mice could be a compensatory feedback mechanism to overcome the excess energy storage following chronic DHT treatment. It is also possible that the decrease in food intake may be a secondary consequence of decreased physical activity; however, in the DHT-treated miR21KO there was no change in caloric food intake despite a similar reduction in physical activity. Interestingly, a possible direct effect of androgens on the regulation of food intake was reported [86]. The loss of this effect in the DHT-treated miR21KO mice indicates an impairment of this potential mechanism and a further energy imbalance in those mice.

To explore the mechanisms involved in increased BAT mass and its associated reduction in EE, we assessed BAT de novo lipogenesis by measuring the protein expression of two FA synthesis markers. Increased lipid deposition in the BAT induces its involution, which is characterized by a reduction in both mitochondrial mass and BAT thermogenic activity [87]; hence, inhibiting de novo lipogenesis could be a potential therapeutic target to prevent the loss of BAT thermogenic capacity. Our data indicate that ACC, the FA synthesis marker, was downregulated by DHT only in WT mice. This effect could be a compensatory mechanism to attenuate excessive adipose tissue expansion by decreasing de novo lipogenesis. Under normal diet conditions, female rats express higher BAT levels of FAS compared to males [88], explaining the reduction in FAS expression in response to androgens in the DHT-treated WT mice in our PCOS model. Moreover, sex-specific differences in BAT FAS expression tend to have opposite expression patterns in response to diet-induced obesity [88]. Excitingly, our miR21KO mice showed an opposite effect of DHT on FA synthesis markers expression, where they have a significant upregulation in BAT FAS protein levels, indicating increased lipogenesis. Notably, FAS inhibitors have been reported to inhibit food intake [89]. This could explain the genotype-specific effect of DHT on caloric food intake, which was only decreased in DHT-treated WT mice and was accompanied by comparable decreases in BAT FAS protein levels.

The role of miRNAs as critical regulators of lipid synthesis and FA oxidation has been reported [90]. Our results indicate that miR-21 inhibits de novo lipogenesis in BAT from DHT-treated mice, as FAS is upregulated by DHT in the absence of miR-21. There have been no studies on the effect of miR-21 on adipose tissue de novo lipogenesis; however, this finding is supported by miR-21's ability to inhibit lipopolysaccharide-induced lipid accumulation and lipid-laden foam cell formation in macrophages [91]. Notably, silencing miR-21 expression reverses the latter effect via targeting TLR-4 signaling [91], which is a known inducer of de novo lipogenesis in the liver [92, 93]. Moreover, lipid deposition and foam cell formation in macrophages can be mediated by de novo lipid synthesis, in addition to the well-known classical mechanism in which foam cells are produced by cholesterol uptake from oxidized LDL [94]. On the other hand, miR-21 was also reported to induce hepatic steatosis and promote the progression of NAFLD [90], implying that miR-21's effect on lipogenesis is tissue- and disease-specific.

The RER, also known as the respiratory quotient, is a useful tool for estimating the fuel source used for energy production based on the number of oxygen molecules required for the oxidation of glucose versus fatty acids. Decreasing RER values trending towards a minimum values of 0.7 indicate that fatty acids are the primary oxidative substrate, whereas increasing RER values towards a maximum value of 1.0 indicate that carbohydrates are the primary energy substrate [95]. In the light phase, the RER was significantly increased by DHT only in WT mice, indicating a tendency towards increased carbohydrate utilization. Moreover, despite no change in RER in the dark phase in WT-DHT mice, miR21KO-DHT mice showed a significant reduction in RER, indicating relying more on lipids as the energy source. The increase in BAT de novo lipogenesis (higher fatty acid synthesis markers expression), decrease in carbohydrate utilization, and increased reliance on lipids as an energy source in our miR21KO-DHT mice, as indicated by the reduced RER, could be explained by an energy surplus caused by an increase in whole-body fat content following 12 weeks of DHT treatment in our mice. This observation is supported by the finding that miR-21 modulation has no effect on cellular lipid content in H9C2 cardiomyocytes under normal nutrient conditions [96]. In the presence of lipid excess, however, miR-21-5p overexpression reduced the lipid-induced increase in cellular lipid content, whereas its suppression increased cellular lipid accumulation and decreased glucose consumption [96].

BAT adipogenesis is critical for BAT structure and thermogenic function. Decreased capacity of compensatory de novo adipogenesis is associated with increased central obesity [97]. Failure of adipocyte differentiation leads to impaired ability of the adipose tissue to expand to accommodate energy surplus leading to insulin resistance and lipodystrophy in which lipids are deposited into multiple organs across the body [98]. Our data indicate that the effect of androgens on the expression of key transcriptional regulators of adipogenesis and adipocytes differentiation, such as adiponectin, PPARγ, C/EBPα, C/EBPβ [99, 99, 99, 99, 99, 99], and MMP9 are modulated by miR-21. Adiponectin, an adipose tissue-derived adipokine, plays a protective role in various physiological conditions including thermogenesis [100]. Adiponectin enhances cold-induced subcutaneous WAT (scWAT) browning [101] and its genetic ablation showed impaired adaptive thermogenic program following cold exposure or high-fat diet -induced obesity in both scWAT and BAT [101, 102], and PPARγ expression downregulation [102]. PPARγ is another adipogenesis marker that plays a pivotal role in adipocyte differentiation, and its deficiency is associated with impaired BAT thermogenesis, lipodystrophy, and a worsened metabolic phenotype [103–105]. Despite preserved adiponectin mRNA and protein expression following DHT in WT mice, its mRNA was downregulated by DHT in miR21KO mice, which also showed significantly lower adiponectin and PPARγ protein levels compared to DHT-treated WT mice. Our data is in accordance with others indicating the potential role of miR-21 in preadipocyte differentiation. MiR-21 overexpression during adipocyte differentiation increases both adiponectin mRNA and protein expression in 3T3-L1 adipocytes [106], and the use of miR-21 mimics or its overexpression induces PPARγ expression in the BAT of obese mice [35] and human adipose tissue-derived mesenchymal stem cells (hASCs), respectively [107]. Among the C/EBPs family members, C/EBPβ enhances the differentiation of 3T3-L1 preadipocytes toward the BAT rather than the WAT phenotype [108]. While one study showed that C/EBPα is required for differentiation of white, but not brown, adipose tissue [109], other studies indicate its critical function in BAT adipocyte differentiation by regulating BAT thermogenic and adipogenic gene expression [110]. Notably, C/EBPβ regulates adipose differentiation by activating the expression of PPARγ and other adipogenic genes, including C/EBPα [111]. Interestingly, C/EBPβ mRNA expression and C/EBPα activation were only reduced in DHT-treated miR21KO mice. Moreover, the increased C/EBPβ activation (evidenced by increased phosphorylation) by DHT in the WT mice was lost by miR-21 genetic ablation, implying that C/EBPα and C/EBPβ are possible indirect miR-21 targets.

Adipose tissue structure and function are maintained with the help of extracellular matrix (ECM), and its remodeling plays a critical role in preadipocyte differentiation and adipocyte growth [112]. The imbalance between matrix metalloproteinases (MMPs) and their endogenous inhibitors, tissue inhibitors of metalloproteinases (TIMPs), is involved in obesity development via modulating adipocyte differentiation and disrupting adipose tissue ECM turnover [113]. Silencing of miR-21, but not miR-224, reduced MMP9 protein levels after cerebral ischemia [114]. Despite other studies indicating that MMP9 inhibition can reduce fat mass and adipose tissue hypertrophy, pharmacologic inhibition of MMP2 and MMP9 reduces preadipocyte differentiation in vitro and reduce PPARγ induction [115, 116]. In addition to its reported regulatory effect on adipogenic differentiation [107], miR-21 can modulate angiogenesis through the modulation of VEGF-A [117], which has a positive effect on BAT thermogenic function [50]. Despite the lack of significant changes in VEGF-A expression by DHT or miR-21 genetic ablation, MMP9 expression was downregulated only in DHT-treated miR21KO mice. Our data suggest that miR-21 function is required for the proper development and differentiation of brown adipocytes and, in turn, its thermogenic function. Our study shows for the first time the effect of miR-21 genetic ablation on BAT adipogenesis in PCOS.

The fatty acid-binding proteins (FABPs) are a family of small cytoplasmic proteins, that are highly expressed in both adipocytes and macrophages and hence play an important role in inflammation related to lipid metabolism [118]. The adipocyte-FABP (also known as FABP4 or aP2) functions as a lipid chaperone that regulates FFAs trafficking and signaling and reduction in its activity or expression is associated with a metabolically favorable phenotype in human and animal models [119, 120]. Our mice did not show any significant changes in FABP4 expression by DHT in either genotype; however, FABP4 basal levels were higher in vehicle-treated miR21KO mice compared to their WT counterparts.

A number of transcription factors are known to play a pivotal role in the regulation of adaptive thermogenesis on the transcriptional level. In addition to its ability to trigger myogenic (*Myf5*^+^) and white adipose tissue precursor’s differentiation into brown and beige adipocytes, respectively, PRDM16 induces the expression of UCP1 and PGC-1α [121, 122]. PGC-1α is another transcriptional coactivator that regulates mitochondrial biogenesis and oxidative metabolism [123]. UCP1 overexpression in white adipocytes can drive adipocyte beiging [123]. CIDE-A is another lipid-droplet-associated protein that transcriptionally regulates UCP1 function [25]. Our data indicate no changes in the expression of either PRDM16 or PGC-1α by DHT in both genotypes. However, despite higher basal CIDE-A levels in miR21KO mice, both the mRNA and protein levels of this transcription factor were significantly downregulated by DHT only in DHT-treated miR21KO mice, which also showed lower CIDE-A protein levels compared to DHT-treated WT mice. Despite a lack of studies on the effect of miR-21 role on the expression of thermogenic targets in PCOS, the use of miR-21 mimics in obesity induced by high-fat diet was associated with upregulation in multiple genes involved in thermogenesis, including PRDM16, PGC-1α, UCP1, and CIDE-A [35].

Thyroid hormones constitute a major mechanism for controlling BAT activity. DIO2 enzyme mediates the formation of the T3 from thyroxine in the BAT [26]. T3 transcriptionally regulates UCP1 and other mitochondrial biogenesis genes including PGC-1α, nuclear respiratory factor 1 (NRF1), and cytochrome c (Cyt c) by binding to its nuclear receptor in brown adipocytes [27, 124–126]. DIO2 activation is associated with increased energy expenditure in BAT [127], and its deletion is associated with impaired BAT thermogenesis following cold exposure despite normal plasma T3 levels [126]. Our data show that UCP1 levels did not change in response to DHT; however, downregulation of DIO2 mRNA levels was observed in both genotypes. Our findings are consistent with those showing that despite a normal plasma T3 concentration and normal basal UCP1 levels, cold-exposed DIO2 knockout (DIO2-KO) mice had impaired BAT thermogenesis. In the same study, brown adipocytes from DIO2-KO mice showed an attenuation in norepinephrine-induced increase in UCP1 mRNA due to impaired cAMP generation [126]. In our study, the effect of DHT on DIO2 expression was similar in both miR21KO and WT mice. Notably, miR-21 positively regulates DIO3 while it has no effect on DIO2 in basal cell carcinoma cells [128].

Steps involved in mitochondrial thermogenesis include the release of FA from lipid droplets by lipolysis followed by its transport to the inner mitochondrial membrane to be involved in β-oxidation, a major step involved in BAT thermogenesis [129]. Long-chain fatty acids (LCFA), constitute a major fraction of fatty acids delivered to target tissues [130]. LCFAs activate UCP1’s heat generation function in the BAT [131]. Elovl3, also known as cold-induced gene (Cig30), is a member of a mammalian gene family that is involved in the biosynthesis of very long chain fatty acids (VLCFAs) in the C20-C24 range and is mainly expressed in liver, skin, and BAT [132]. In addition to playing a critical part in the development and recruitment of brown adipocytes, Elovl3 is also substantially elevated to function in thermogenesis after exposure to cold [49]. The levels of Elovl3 are correlated with the oxidative capacity of FA in the brown adipocytes [133]. Our data indicates similar downregulation of Elovl3 gene expression in the BAT of both miR21KO and WT genotypes, indicating another mechanism of impaired thermogenic responses in PCOS independent of miR-21 levels. FA release by lipolysis is under control of perilipin, a protein family that coats the lipid droplets of adipocytes and act as barrier against the lipolytic action of lipases [134, 135]. Perilipin-1, the predominant isoform of perilipin expressed in white and brown adipocytes [136], was not modified by DHT treatment or miR-21 genetic ablation.

The carnitine palmitoyltransferase 1 (CPT1) is the rate-limiting enzyme in mitochondrial FA oxidation as it facilitates mitochondrial FA transport for β-oxidation. CPT1B is the most abundant isoform in BAT; however, increased CPT1A isoform expression in brown adipocytes is also reported to increase FA oxidation, lipolysis, and UCP1 activity [137]. Our data show that both CPT1A mRNA and CPT1B protein expression were upregulated by DHT in WT mice, and this effect was lost by miR-21 genetic ablation. These effects could be attributed to the attenuation in DHT-mediated upregulation in AR in DHT-treated miR21KO mice. Notably in male mice, AR is involved in energy homeostasis as its blockade results in lower energy efficiency and fat metabolism following chronic exercise by suppressing CPT1 expression in the skeletal muscle [138]. Therefore, the increase in CPT1 in our DHT-treated WT mice could be a homeostatic mechanism to maintain energy balance in response to obesity by burning more FAs.

The terminal and rate-limiting step of mitochondrial respiration is the reduction of molecular oxygen to water via cytochrome c oxidase (COX) using cytochrome c as substrate [139]. COX, a complex of the mitochondrial respiratory chain, is a dimer consisting of 13 individual subunits per monomer [140]. We assessed the expression of multiple COX subunits of significant expression and functional activity in the BAT, including Cox4i1, Cox4i2, Cox7a1 [141], and Cox8b [142]. Our data indicate that although DHT did not induce any significant changes in the assessed COX subunits in WT mice, COX4 protein expression was significantly downregulated by DHT in miR21KO mice, indicating less mitochondrial activity and hence BAT thermogenic function regulation by miR-21 genetic ablation. Several miRNAs are reported to modulate electron transport chain components, including COX4 in a negative or positive manner [46]. While miR-338 downregulates COX4 leading to reduced mitochondrial activity in sympathetic neurons [47], knocking down miR-182 and miR-203 leads to lower COX7 and COX8 expression in primary brown adipocytes [48]. Our data is the first to indicate a possible regulatory effect of miR-21 on COX4 expression in the BAT in a PCOS model.

Adaptive de novo lipogenesis inhibition and increased expression of adipogenesis and thermogenic markers is a mechanism for achieving energy balance in conditions of obesity and energy surplus. In our PCOS model, androgen-induced obesity was associated with the downregulation of lipogenesis markers, increased adipogenesis (higher C/EBPβ activity), and upregulation of CPT1A and CPT1B key thermogenesis and mitochondrial biogenesis markers. MiR-21 genetic ablation in DHT-treated mice, on the other hand, increased de novo lipogenesis while inhibiting BAT differentiation, as evidenced by a significant decrease in mRNA and/or protein expression of key BAT adipogenic genes such as adiponectin, PPARγ, and C/EBPβ, which was accompanied by downregulation of CIDE-A and COX4 thermogenic markers. In addition, the adaptive increase in CPT1A and CPT1B was lost upon miR-21 genetic ablation. Several miRNAs have been reported to regulate brown fat differentiation and thermogenic function, either positively or negatively. However, this is the first study to investigate the role of miR-21 in altered thermogenic responses caused by excess androgens in a PCOS model, with miR-21 genetic ablation abolishing androgen-mediated beneficial effects on BAT thermogenic markers expression. The latter effect could be explained in part by attenuating androgen-mediated BAT AR upregulation in DHT-treated miR21KO mice. In males, AR activation has been shown to have beneficial thermogenic functions [138]. On the other hand, androgens have been reported to have deleterious effects in WAT in females. Androgens, via AR activation, inhibit adipogenesis in non-obese women's human adipose stem cells (hASCs) [143], which is postulated to promote ectopic lipid deposition and lipotoxicity, and its administration to women inhibits subcutaneous abdominal adipose lipolysis by inhibiting hormone-sensitive lipase [144]. It is possible that the negative obesogenic effects of long-term hyperandrogenemia on total body fat mass are mitigated in part by the positive AR thermogenic action in BAT. This conclusion is supported by data demonstrating disease and cell-specific differential regulation of AR and miR-21. While miR-21 downregulates AR leading to a deleterious effect on cardiac fibrosis [77], in prostate cancer, miR-21 increases AR protein expression and is involved in tumor pathogenesis [78]. Future studies using depot-specific adipose tissue modulation of miR-21 levels are needed to identify specific androgen effects on WAT and BAT, which could be a novel therapeutic approach for the treatment of PCOS-associated metabolic derangements.

## Perspectives and significance

Our findings strongly suggest that miR-21 function is required for normal BAT adipogenesis in addition to its roles in thermogenic machinery and whole-body homeostasis. Moreover, our findings suggest that miR-21 plays a critical role in the molecular adaptive responses of BAT to hyperandrogenemia in PCOS. Furthermore, increased circulatory miR-21 levels in PCOS could be a compensatory mechanism that helps mitigate systemic androgen-mediated metabolic dysfunction, providing a new theoretical foundation for the development of miRNA-based therapeutics for PCOS-related adipose tissue dysfunction.

## Data Availability

The datasets used and/or analyzed during the current study are available from the corresponding author on reasonable request.

## Abbreviations

ACC: Acetyl-CoA carboxylase
AR: Androgen receptor
BAT: Brown adipose tissue
C/EBP: CCAAT/enhancer-binding protein
CIDE-A: Cell-death inducing DNA fragmentation factor-like effector A
COX: Cytochrome c oxidase
CPT1: Carnitine palmitoyltransferase 1
DHT: Dihydrotestosterone
DIO2: Type 2 deiodinase
EE: Energy expenditure
FABP: Fatty acid-binding proteins
FAS: Fatty acid synthase
FFA: Free fatty acids
LCFA: Long-chain fatty acids
MMP: Matrix metalloproteinases miRNAs microRNAs
miR21: MiRNA-21
PCOS: Polycystic ovary syndrome
PPARγ: Peroxisome proliferator-activated receptor gamma
Ppargc1a or PGC-1α: Peroxisome proliferative activated receptor gamma coactivator 1 alpha
PRDM16: PR domain containing 16
RER: Respiratory exchange ratio
TIMP: Tissue inhibitor of metalloproteinases
UCP1: Uncoupling protein 1
VEGF-A: Vascular endothelial growth factor-A
WT: Wild-type
WAT: White adipose tissue
